# Exercise and physical activity in asylum seekers in Northern England; using the theoretical domains framework to identify barriers and facilitators

**DOI:** 10.1186/s12889-018-5692-2

**Published:** 2018-06-19

**Authors:** Melanie Haith-Cooper, Catherine Waskett, Jane Montague, Maria Horne

**Affiliations:** 1 University of Bradford, Bradford, UK; 2 University of Leeds, Leeds, UK

**Keywords:** Asylum seekers, Physical activity, Exercise, Theoretical domains framework

## Abstract

**Background:**

Many asylum seekers have complex mental health needs which can be exacerbated by the challenging circumstances in which they live and difficulties accessing health services. Regular moderate physical activity can improve mental health and would be a useful strategy to achieve this. Evidence suggests there are barriers to engaging black and minority ethnic groups in physical activity, but there is little research around asylum seekers to address the key barriers and facilitators in this group.

**Methods:**

A two stage qualitative study used semi-structured interviews underpinned by the Theoretical Domains Framework. The interviews were conducted in voluntary sector groups in four towns/ cities in Northern England. Purposive sampling recruited 36 asylum seekers from 18 different countries. Interviews were audio recorded, transcribed verbatim and subject to framework analysis. Stage two involved a nominal group technique with five key stakeholders including asylum seekers and those that work with them. They followed a four stage process to rank and reach consensus on the key barrier to undertaking physical activity/ exercise that could be addressed locally through a future intervention.

**Results:**

A number of barriers and facilitators were identified including a lack of understanding of the term physical activity and recommended levels but knowledge of the health benefits of physical activity/ exercise and the motivation to increase levels having engaged with activities back home. Living as an asylum seeker was considered a barrier due to the stress, poverty and temporary nature of living in an unfamiliar place. The outcome of the nominal group technique was that a lack of knowledge of facilities in the local area was the prevailing barrier that could be addressed.

**Conclusions:**

Public health practitioners could develop interventions which capitalise on the motivation and knowledge of asylum seekers to encourage an increase in physical activity which may in turn reduce the breadth and depth of mental health needs of this group.

## Background

In 2016, an estimated 65.6 million people worldwide were forced to flee their homes and of these 2.8 million claimed asylum in another country [1]. This was most commonly a country adjacent to home, however, 1.2 million people claimed asylum in European countries [2] with 30,747 in the UK [3]. On arrival in the UK, asylum seekers are generally housed in initial accommodation centres for 2 weeks and are then ‘dispersed’ to different areas across the country to await a decision on their asylum claim, which in some cases can take several years. They are generally provided accommodation, are not allowed to work, and receive a very small amount of cash for living. In 2017 in West Yorkshire over 3000 asylum seekers were receiving Home Office support [4], however, total numbers are unknown. For example, refused asylum seekers are excluded from these statistics, receiving no Home Office support [5]. Their asylum claim has been unsuccessful and they may be awaiting an appeal or be at the end of the asylum system but may be frightened or unable to return home.

Many asylum seekers have complex mental health needs. Symptoms may be triggered by trauma and violence experienced back home and a difficult journey to the host country leading to anxiety, depression or post-traumatic stress disorder [6, 7]. This can be exacerbated in the host country by hostile public attitudes around asylum [8] reinforced through negative media reporting [9, 10]. This can lead to the marginalisation and social isolation of asylum seekers and on occasions, discrimination and violence [11, 12]. The asylum process, not being allowed to work, living in poverty and the uncertainty around the future can lead to a further deterioration of mental health [13–16].

In the UK, although asylum seekers are entitled to NHS services, they can experience barriers to accessing these such as not understanding the health care system [17]. They often rely on voluntary sector services which are unable to cope with demand [6]. Consequently, their mental health can deteriorate further leading in some cases to crisis points and emergency hospital admission This has avoidable economic as well as human cost by ensuring strategies to crisis prevention [18]. This is particularly important in this vulnerable group.

Mounting evidence supports the beneficial effects of regular moderate intensity physical activity to improve mental health with international commitments to increasing physical activity levels amongst the general population, including minority ethnic groups [19–22]. Current recommendations are that adults should undertake at least 150 min per week of moderate-intensity aerobic activity which slightly raises the heart rate but the adult can still talk [20]. This can be unplanned activity, with small shifts in behaviour such as walking faster or increasing the intensity of housework can lead to improvements in health and well-being [22]. In contrast, exercise, is planned and structured, undertaken with the aim of improving health. Vigorous exercise has mental health benefits beyond those of moderate physical activity being associated with less stress, pain, insomnia and depression [23–26].

Evidence suggests that there are barriers to engaging black and minority ethnic groups in physical activity in the UK and more widely [27–30]. Cultural differences appear central to many barriers such as different understandings of physical activity and health causation and the role of the individual within the community [28]. Also, the stigma around exercising in public or removing clothes [29]. However, very little evidence could be found related to asylum seekers. An Australian study [31] focused on exploring the challenges of living in Australia with the use of physical activity as a coping mechanism but no studies could be found examined how behaviour could be changed in asylum seekers through addressing perceived barriers and facilitators to engaging with physical activity and/or exercise. This could be a useful strategy to help them cope with a difficult life and improve their mental health. However, asylum seekers are a heterogeneous group, originating from diverse areas of the world, with different religious and cultural backgrounds [6] and it is important to consider how this could influence perceptions of physical activity and exercise in this group. Consequently, this study aimed to address the following objectives:

1. What are the barriers and facilitators that influence uptake and adherence of physical activity behaviour among asylum seekers in the UK?
2. How can the findings be used to develop an intervention to address the key barriers and facilitators, in preparation for piloting among asylum seekers?

## Methods

### Study design

This study adopted a two stage qualitative design. Stage one used semi-structured interviews underpinned by the Theoretical Domains Framework of behaviour change [32] to address the first research question. This framework is designed to facilitate the integration of theoretical approaches to behaviour change interventions. Eleven domains that can facilitate or hinder health behaviour aligned well with the focus of this study. The excluded domain related to social/professional role and identity which did not appear appropriate in this context as the behaviour change did not relate to a professional or organisational context. For each of the 11 possible domains that could act as facilitators or barriers, the researchers developed several interview questions (Table 1) which explored factors that might influence asylum seekers’ behaviour in relation to undertaking physical activity and exercise. The first series of questions related to knowledge and after asking about understanding of the terms moderate intensity physical activity and exercise, a definition was provided to facilitate the questions for the remainder of the interview.

**Table 1:** Interview schedule using the Theoretical Domains Framework [32] (Goes with section on study design)

Behavioural change domain	Interview questions to explore the domain
Knowledge	• What does moderate intensity physical activity mean to you? • What does exercise mean to you? • Do you know how much PA is recommended for maintaining good health? If participant does not have the knowledge, provide the recommended definition of moderate intensity physical activity and definition of exercise
Skills	• Would you know how to do moderate intensity PA? • How easy or difficult do you find doing moderate intensity PA? • Would you know how to exercise to improve your health? • How easy or difficult do you find doing exercise?
Beliefs about capability	• How confident do you feel about doing 150 min of moderate intensity PA per week? • Back home did you have any problems being able to undertake moderate intensity PA? • Now do you have any problems undertaking moderate intensity PA? Probe: What would help you with these problems? • Do you think you could carry on doing150 min of moderate intensity PA if you started? • Did you have you had any problems exercising back home? • Do you have any problems now undertaking exercise? • Do you think you could continue to exercise if you started?
Belief about consequences	• What do you think will happen increase your PA levels to moderate intensity? • What do you think will happen if you don’t increase your PA? • What do you think are the advantages of doing regular moderate intensity PA? • What do you think are the consequences of not doing regular moderate intensity PA? • What do you think will happen if you start to exercise? • What do you think will happen if you don’t start to exercise? • What do you think are the consequences of not doing regular exercise?
Motivation and goals (intention)	• How much do you want to do more moderate intensity PA? • Are there other things that you think are more important than doing more PA? • How much do you want to do more exercise? • Are there other things that you think are more important than doing exercise/?
Memory, attention and decision processes	• Is exercise something you would normally do? • Probe: If yes, how do you make sure you do your exercise? • What would stop you from doing your exercise? • Would you remember to do 150 minutes of moderate intensity PA over the week?
Environmental context and resources	• Consider how you are living at the moment: • What things make it easy for you to do more moderate intensity PA? • What things make it difficult for you to exercise/do more moderate intensity PA? • Probe: access, finances, time • What things make it easy for you to exercise? • What things make it difficult for you to exercise PA? • Probe: access, finances, time
Social influences	• How would the people that you live and socialise with help you • or make it difficult for you to do more moderate intensity PA? • Prompt: family, peers, social groups • Do you see other people exercise around you? • If yes, does that influence you?
Emotion	• When you do more moderate intensity PA how does it make you feel? • Does this feeling make it easier or harder to do more moderate intensity PA? • When you exercise how does it make you feel? • Does this feeling make it easier or harder for you to exercise?
Nature of the behaviour	• What do you do/what do you think you need to do to before you do more moderate intensity PA regularly? (individual/community/environment) • What do you do/what do you think you need to do to before you can do more exercise? • Thinking about your life at the moment, how do you think this affects you being able to undertake PA? Probe: explain why • Thinking about your life at the moment, how do you think this affects you being able to exercise? Probe: explain why

Stage two used a nominal group technique to develop consensus in prioritising the findings from the interviews and inform future intervention development to address the second research question. In this paper, this is reported following the findings from stage one.

### Stage 1: setting, participants and data collection

After receiving ethical approval, voluntary sector refugee networks were used to identify local support groups that could be targeted to access potential participants. Group leaders were approached and a member of the research team attended a group session. An initial pilot group interview involving three researchers to test the interview schedule and ensure consistency in the way the questions were approached at subsequent interviews. Each interview started with demographic questions then broad, open-ended questions followed by an increasing focus on specific issues based on the Theoretical Domains Framework (Table 1). The interviews took place in voluntary sector groups in four towns/ cities in Northern England.

Purposive sampling was used to recruit participants, aiming for maximum variation reflecting diversity in terms of gender, age, country of origin and cultural and religious background. After receiving informed consent, a total of 36 participants were interviewed in small groups (excluding one participant due to none attendance of other participants) for mutual support and peer interpreting. All participants spoke English as an additional language though their abilities varied. The group leaders assessed the ability of the participants as a group to provide language support for each other when required. Most of the venues attended were ‘conversation clubs’ where participants were attending to improve their English language skills.

All interviews were audio recorded, relaying responses through an interpreter when required. Participants were asylum seekers receiving Home Office support and awaiting a decision or refused asylum seekers awaiting an appeal. Refugees were excluded as they may experience different barriers and facilitators following a successful asylum decision. All potential participants were approached on the day and the researcher read an information sheet about the study. If they agreed to participate, participants were taken to a private area to conduct the interviews. Informed verbal consent was obtained by speaking into the audio recorder at the beginning of the recording. This method of obtaining informed consent was approved by the ethics panel to avoid any fear associated with the participant providing a signature. Participants were also reassured that the researchers had no links with the Home Office, that care would be taken to ensure confidentiality and anonymity and that they had the right to withdraw. Audio recordings were transcribed verbatim and each transcript reviewed by the interviewer to ensure accuracy.

### Stage 1: data analysis

The data from the interviews were subject to framework analysis [33] using the theoretical Domains Framework as a coding framework [32]. Three investigators (ML, JM, CW) read and re-read all the transcripts independently and coded the data, then combined codes into sub themes and allocated these along with direct quotes from participants, to one of the 11 theoretical domains. For example, the statement “*for me, going on in my life at the moment, sometimes you just stay in bed, to wake up from that bed is really hard, it’s like you..not to move…”* was coded into the into the domain ‘Capabilities’ with the code description of ‘Stress and being an asylum seeker’. Regular discussions took place to resolve disagreements and reach consensus and an audit trail was produced by keeping a record of coding decisions. To further ensure analytical rigour, a second iteration of the coding process was performed, with a re-review of the transcripts to identify any important quotes or codes/domains missed or misallocated. In addition one author (MH) a public health academic, provided a critique of the analysis and interrogated the coding to ensure a rigorous and defensible coding of the data into the relevant domains.

## Results

The 36 participants were a particularly diverse group originating from 18 different countries, with an age range from 18 to 59 and over half were female (Table 2).

**Table 2:** Demographic findings

Gender	Home country	Age	Religion
F	Iran	30	Muslim
F	Iran	39	Muslim
M	Syria	28	Muslim
M	Iran	37	Christian
M	Iran	62	Christian
F	Malawi	59	Christian
F	India	29	Sikh
M	Sudan	37	*
M	Libya	47	Muslim
F	Bolivia	29	Christian
F	South Africa	39	Christian
F	Pakistan	30	Christian
F	Sri Lanka	27	Christian
F	Guinea	20	Christian
F	Syria	18	Muslim
F	Syria	*	Muslim
F	Ethiopia	36	Orthodox Christian
F	Syria	45	Muslim
F	Ethiopia	*	Muslim
F	Eritrea	26	Muslim
M	Syria	27	Muslim
M	Sudan	33	Muslim
M	Eritrea	27	Pentecostal
M	Eritrea	21	Pentecostal
M	Eritrea	26	Pentecostal
M	Sudan	27	Muslim
M	Iraq	*	*
M	Iran	52	*
M	Kashmir	43	Muslim
F	Eritrea	28	Christian
F	Eritrea	29	Christian
F	Eritrea	32	Christian
F	Eritrea	36	Christian
F	Nigeria	46	Christian
M	Zimbabwe	45	Christian
M	Zimbabwe	50	Christian

*Data not known or not shared with researchers

The findings highlighted a number of codes in each domain that could be considered a potential barrier or facilitator to asylum seekers undertaking moderate intensity physical activity and/or exercise (Table 3). These are discussed below in relation to the domains of the Theoretical Domains Framework. There was an overlap of themes with the domains, therefore only eight are reported below. No data emerged relating to the domain around emotion. The quotes are labelled with the town/city where the interview took place (L, H, W or B), whether the participant identified as a man or woman and a participant number for that context.

**Table 3:** Fitting the codes into the Theoretical Domains Framework

Behavioural change domain	Description of codes
Knowledge	English as an additional language Lack of understanding of recommended levels of physical activity
Skills	Skills from ‘back home’
Beliefs about capability	Stress and ‘being an asylum seeker’ Mental and physical health Varying confidence levels
Belief about consequences	Impact on physical and mental health
Motivation and goals (intention)	Competing priorities Good motivators
Memory, attention and decision processes	Living in limbo
Environmental context and resources	Living in poverty Living in a foreign place The weather
Social influences	Housemates and family influences Cultural and social factors back home

### Knowledge

None of the participants understood the meaning of the term ‘physical activity’. Generally, participants suggested this related to doing exercise but less intensely, in some cases just walking. Even after receiving a definition of physical activity and the difference between this and exercise, some participants questioned the use off the term. Although some grasped the difference between this and exercise, others still appeared not to understand:

*WW1 Yeah, I like physical activity because I’m exciting to do, to make something to enjoy to some group, to conversation, or making something or doing like art or something like that, I like.*

Others discussed the terms interchangeably throughout the interview.

*BW5 You know even housework also is one exercise. Also cleaning, cooking, you know ironing, this also one exercise. And also not taking transport also, this was exercise, you know from my house going to town, this was exercise, yeah.*

No participants understand the term moderate intensity. However, some were aware that they needed to increase their heart rate and feel warm for the physical activity to be beneficial to their health. Participants also varied widely in their understanding of how much moderate intensity physical activity is recommended to stay healthy. This varied from 2 to 3 times a week to every day:

*HM1 I think daily you have to make at least three hours daily, physical activities, that’s mean three times seven is 21, 21 hours weekly.*

### Skills

It became apparent that many participants; both male and female had undertaken sport before leaving their home country and were skilled in activities such as bodybuilding, football, cycling, water sports, swimming, volleyball and basketball:

*LW1 In my country, we do a lot of exercise. P.E. is a quite big subject in the school, and it’s very, like, competitive.*

There were participants who currently used their skills, accessing sport in the UK. However, others didn’t know how to and some participants wanted to learn new skills including swimming and riding a bicycle but again felt that they couldn’t due to other barriers discussed in the other domains:

### Capabilities

‘Living as an asylum seeker’ was the main influence on participants’ perceptions of their capability to undertake regular physical activity and exercise. The asylum process was considered an emotional roller-coaster and at times when stress associated with their status was more intense, this created difficulties engaging with everyday life and therefore concerns about being fit and healthy were not considered a priority:

*LW2 …for me, going on in my life at the moment, sometimes you just stay in bed, to wake up from that bed is really hard, it’s like you are really straining yourself. So with the stress you have, you just prefer to stay on that bed. Not to move…*

However, some participants used physical activity as a way of improving their wellbeing

*LW3 …the doctor prescribed me antidepressants, but I am not using, instead of this if I think something I just go for a walk, and I almost, I walk almost five miles a day, so…*

Physical health conditions were felt to influence capability to undertake physical activity and/ or exercise. This included back pain, heart conditions, breathing problems, diabetes and also sports injuries. Some participants identified that they were aware physical activity could improve their physical health condition

*LW4 I’m pre-diabetic, so, I know physical exercise is supposed to help, so, I don’t want to become diabetic next year.*

A number of participants felt confident in their capability to undertake the recommended level of physical activity and exercise.

*LW8 …I was shy when I go to gym, I was started the gym, and then, but at the, after 2, 3 days I was have full of confidence so I don’t care what exercise they are doing the people, I do whatever I want, and so if you’re building confidence you don’t think about anything, you think about only yourself, so people they don’t affect exercise actually.*

Other participants identified that they did not feel confident, however this related more to exercise than physical activity.

*HW1 They are not easy at all. I don’t even know where to start. Yeah, it’s quite difficult for me.*

### *C*onsequences

Participants appeared to understand the importance of physical activity and exercise and the health consequences of not engaging in these. They related a sedentary lifestyle to the risk of stroke and heart attacks and also generally needing medical treatment. They discussed how they felt physically when they did not engage with physical activity and/ or exercise:

*HM1 Sure it will affect all my inside in my body, also my body activities, that’s like not fit at all, I will feel not fit and it’s easy to exhaust after doing a small job, I can feel it.*

Some female participants focused on weight gain as a consequence of not exercising and the need for a balanced diet as well

*LW10 It’s not just doing it, you have to like check your input as well, if you eat a balanced diet because if you are doing it and not eating the food right that wouldn’t be wise, it’s like there’s no point, if you are balancing your diet and doing that exercise health-wise you will benefit from it.*

### Motivation

Most participants felt motivated to increase their level of physical activity and exercise. However, a number of participants felt that they had competing priorities which did make it difficult to maintain motivation. This often related to self-development to help the process of settling in the UK

*BM 8 (about BM7) He want to study English only.*

Motivation depended on the type of physical activity and whether this was perceived as enjoyable. It was suggested that the support of a coach and a structured exercise programme would increase motivation:

*LW1 Oh, I think doing a structured exercise, it makes you gain a lot of discipline, because you have to stick to a routine, so that will actually affect everything else you do, because you stick to a routine. So discipline I think that is good.*

Some participants had continued to exercise whilst living in the UK though the amount and degree was highly variable. Daily physical activity rather than exercise was common for most of the participants. This included gardening, shopping, caring for children, walking and running for the bus.

### Memory, attention and decision processes

‘Living as an asylum seeker’ and the uncertainty for the future was believed to affect the conscious decision of whether to start exercising if you were likely to be moved or deported very soon. Feeling settled and secure where you are living were considered important facilitators in the decision process

*LM2 …if you were then to start an exercise at a gym….you need to know that’s the, you’ll be able to do it. As well as you doing your physical exercise today, knowing that maybe after two weeks or maybe three days, it’s not going to be happening…you wouldn’t even know what would happen to you in two weeks’ time, three weeks’ time, yeah. It’s just like staying in a kind of a limbo. The thing is, if suppose you’re an asylum seeker, you don’t know when you may be detained. You don’t know when, maybe, you may be deported, you don’t know when you may be called.*

In contrast, many participants felt the type of work and family responsibilities I n their country of origin involved significant physical activity. Consequently, they did not need to make a conscious decision to be active, it was the norm:

*LW9 Yeah in the house - water, if you need water not like you have the tap at the house you know, you get it, out of the house and you have big bucket…and it’s heavy you know.*

### Environmental context and resources

Most participants discussed how living as an asylum seeker meant they had very little money; creating a barrier to accessing activities, such as gym membership or the equipment and clothing considered necessary to undertake the activity:

*LM1Yeah, I’ve got some friends, they do play football….They actually play at the weekends, in some tournaments…I like football anyway…I cannot go with my trousers, you know, or my sandals, I can’t. I need to make sure I’ve got all the right, I need money*.

Accessing activities was a low priority when their family was hungry. This would appear more pronounced for a refused asylum seeker without recourse to public funds whose priority is spending time finding where the next hot meal would be coming from:

*LM2 …You don’t have any food. So you kind of live on people who are willing to give you food. So, sometimes it might be, the life where you have got to be moving from place to place where they’re dishing out food, so, it’s [laughs], it’s very weird way of living…“Monday, I’m at, maybe Tuesday, I’m at another place”, because that’s where you can get the hot meal…*

Even when resources such as free gym access were being offered to asylum seekers timing was a barrier, especially fitting around children.

A number of participants talked about how they walked everywhere, not having money for the bus fare. This was considered a barrier to accessing any further physical activity or exercise due to time constraints and also tiredness:

*BM2 they offer two hour in one day, Monday….on Wednesday a free gym, but it’s too far from my…it’s take me one and a half hour walking to this gym, so it’s not worth it because when I go to that I already exhausted.*

However, there was an acknowledgement that walking could be undertaken without needing any money and could be considered a facilitator in this context.

‘Living as an asylum seeker’ frequently involved being dispersed to a new town or city with little knowledge of the area. This appeared a barrier to many participants undertaking physical activity and exercise.

*LW1 …you should, like, have the option to say, “I’m going to do this going there,” so you will know where to go, because if you want to do something but you don’t know where to go, or who to go with, you’re just going to say, “Oh, tomorrow.”*

For example swimming was a popular choice for some asylum seekers, having swam at home but not knowing where to go to swim here. The weather was also considered a barrier to a number of participants

*BM2 For me sometimes, the weather. If I want to go to the gym and the gym is far…Yeah, cos you can come outside, very tired and if weather is cold, coming cough.*

This is in contrast to experiences back home where many participants spoke of how the natural environment around them meant that exercise opportunities were easily available. This included swimming in rivers and the sea, weight lifting with cement and stones, farming and mountain climbing:

*WM3 ……..there is a mountain in north of Tehran, we climb to 3,000 metre in the peak of this, every, every week that was routine. Fridays, 3,000 metre for one bag and I think that was the best for me, in winter, all the seasons, every week so.*

Having a family was considered time consuming and a barrier to undertaking physical activity. For participants, this barrier appeared to be exacerbated by a lack of family support caring for children whilst physical activity or exercise was undertaken.

### Social influences

In the UK, some participants lived with family, others lived with housemates. They identified that their friends and family, influenced their levels of physical activity. This could be in a negative way:

*LW2 I live with my husband and my four children, so if they are around, to be honest with you, they make me more lazy, because the more I sit down, “Give me tea, give me that,” but if they’re not around, I know I have to stand up and do that physical for myself…*

Others did undertake physical activity with their friends and family, this included playing football and walking together:

*LW4 Like, two weeks ago, I received a letter and I was really stressed that they actually opened my file, so my friend said, “Let’s go for a walk,” and she took me to (place),* via *the canal, it was beautiful. It killed me, because you know, she said, “It’s only, like, five miles,” which in my head didn’t seem very far, but when I actually, after eight miles, I felt the burn, but that weekend I slept, which was brilliant. So that calmed me down.*

Some female participants discussed how gender and social and cultural factors back home had been a barrier to them undertaking physical activity and exercise there:

*LW4 So in Saudi Arabia, I found it very difficult, because as a woman I wasn’t allowed to go out, so barely, I was in a flat, no physical activity, and it’s not very encouraged in schools either, so I don’t, I have never played a sport.*

This included in country segregation:

*LW2 In South Africa, as you know, it was apartheid for a very long time, as blacks, and where I’m from, the sort of, like, ghetto, Soweto, I wouldn’t have been allowed to go to town and join white culture to do exercise at all.*

For these participants, arriving in the UK may be a facilitator for women to undertake physical activity. However, their cultural norms may still influence their ability to engage with physical activity and/or exercise.

### Stage 2: nominal group technique

The method first described by Delbecq et al. [34] was followed. Five stakeholders with different backgrounds were approached to provide a broad bottom up perspective to consensus building. The group comprised of a range of individuals with experience of working with or on behalf of asylum seekers and refugees in both supportive roles and as facilitators of physical activities for local groups. Some had been through and were going through the asylum process themselves. With a growing emphasis on the importance of service users as ‘experts by experience’ this was appropriate. We also had a local Public Health practitioner. In the discussions undertaken as part of the process they offered valuable insights and knowledge which appeared to inform their prioritisation.

The four step process was undertaken with a group facilitator presenting the key findings from stage one (Table 4) as choices. Firstly, participants individually selected the barrier or facilitator, which they believed was most important to address at a local level. This was recorded and a group discussion followed until consensus was reached as to what were the five most important options that could be addressed. These were then presented to the group for participants to rank with five being the most important and one being the least (Table 5). The completion of the final rankings explicitly reveals the distribution of individual responses in a statistical form, offering a fuller picture of the process and how consensus was achieved [35]. As can be seen in Table 5, with a total of 20, the most important priority to enable asylum seekers to undertake physical activity and exercise was seen as access to knowledge about opportunities to do so in the local area. This was discussed further to identify stakeholders’ perceptions of the focus for a future intervention to address this barrier.

**Table 4:** Summary of key findings

Key barriers	Key facilitators
Capabilities: Stress associated with being an asylum seeker	Stress drove activity
Decision process: Living in limbo	Feeling settled and secure
Resources and environment Lack of money for food and equipment Lack of knowledge of facilities in local area The weather Time Lack of childcare	
Social	
Exercising With friends/ family Alone in the UK Cultural influences	Exercising With friends/ family
Knowledge and skills	
The phrase ‘physical activity’ and recommended levels	Levels of exercise and physical activity back home
	Understanding consequences Impact on physical health Weight gain Impact on mental health

**Table 5:** Ranking and final scores for the most popular options

	Participant 1	P2	P3	P4	P5	Total
A lack of money	3	5	2	3	3	16
B lack of knowledge of local area	5	4	5	2	4	20
C living in limbo	2	3	3	1	2	11
D lack of food	1	1	1	5	1	9
E cultural influences	4	4	4	4	5	19

## Discussion

This qualitative study has provided valuable insights into the barriers and facilitators asylum seekers face when engaging with the recommended levels of physical activity. Participants lacked understanding of the term physical activity and were not aware of recommended levels. They did however understand the importance of physical activity and exercise in relation to good physical and mental health. Most participants appeared skilled in a number of sports and back home physical activity was considered the norm. The level of current activity varied between participants, some found ‘living as an asylum seeker’ affected their decision to engage with physical activity and/or exercise due to competing priorities, the temporary nature of their living and their mental and physical health. The environment was considered a barrier, living in poverty and not knowing how to access facilities where they were living. Family and friends influenced activity levels some in a positive and others in a negative way. However, many participants did engage with physical activity and exercise, others were motivated to. Cultural barriers back home were discussed by women but not in the context of where they were living now. The outcome of the nominal group technique was that a ‘lack of knowledge of facilities in local area’ was the prevailing barrier which could be addressed at a local level through a future intervention.

Some of our findings reflect previous work around barriers to physical activity in black and minority ethnic groups [27, 28] and migrant populations [30, 36]. Koshoedo et al.’s meta-ethnographic analysis of 14 studies developed four main concepts which some of our findings support [28]. Personal barriers around being an asylum seeker such as competing priorities and time pressures, distance to travel and not knowing how to access facilities were all discussed in our study. However, cultural differences is a major concept in previous literature including Koshoedo et al. [28], work a systematic review around migrant populations [36] and an American study involving South Asian women [37]. In our study, cultural barriers was only mentioned by some women related to back home and were not discussed in the context of living in the UK. Interstingly, the nominal group technique ranked culture as the second most important barrier to address. This is in contrast to the participants in the interviews who saw it as less of a priority. It cannot be assumed that cultural barriers don’t exist but it would appear that other aspects of living as an asylum seeker were considered more important.

Our study demonstrates that if the barriers to undertaking physical activity are addressed, asylum seekers appear well motivated to increase their levels of physical activity. This is in contrast to previous work that has found motivation to be low [27, 30, 31]. A study focusing on Asylum seekers in Australia [31] found that nearly half of participants were not motivated to undertake physical activity and restrictive Government policy was blamed for this. The poverty associated with living as an asylum seeker was a barrier in our study but this did not appear to affect motivation. It is acknowledged that level of financial support offered to asylum seekers varies between countries and that this is an important influence on the decision to engage with physical activity. Basic needs, for example overcoming hunger must be considered when developing physical activity interventions in this group. In addition, there must be no financial cost associated with an intervention due to competing demands on funds.

Our findings are relevant for public health practitioners. Interventions could be developed that capitalise on the motivation to increase physical activity levels or undertake structured exercise to benefit physical and mental health. Interventions could relate to educating asylum seekers about increasing levels of physical activity through basic modifications to lifestyle such as walking faster or increasing intensity when undertaking housework. This would ensure these changes are accessibility for a group with few resources. Interventions could also include working with service providers to offer structured exercise programmes, with no financial implications. This could include the opportunity for team sports such as football and the ‘borrowing’ of appropriate resources such as footwear.

### Strengths and limitations

To develop theory-based interventions, the major determinants of exercise and physical activity behaviour must be identified [38]. Within this study, the use of small group interviews allowed us to explore in detail participants’ perceptions of their barriers and facilitators to undertaking recommended levels of physical activity using the theoretical domains framework. The sample size was sufficient to recruit a heterogeneous group. However, accessing participants through asylum seeking support groups may have excluded a population that does not engage within the community and are possibly more isolated and would benefit from increasing their physical activity levels. In addition, some participants had limited English language skills and although they were able to hold conversations, the ability to communicate on specific health related topics may have limited the exploration of some of the issues raised in more depth. Nevertheless the heterogeneous nature of the findings could be transferable to asylum populations in other areas of the UK and abroad.

The credibility of the nominal group technique could be questioned with such a small number of participants and not include multiple iterations which is considered as an advantage of the method due to the possibility to reflect and change mind sets [39]. It is possible that consensus was reached quickly due to the size of the group. However, participants did represent key stakeholders and the process was carefully followed with two facilitators (MC, JM). Time was allowed for detailed discussions prior to consensus being reached and all participants contributed equally to the process. Consensus was reached on what was perceived to be a barrier that could be addressed at a local level by public health practitioners. This was a pragmatic process leading to the de-prioritisation of other important barriers such as a lack of money and food. These important barriers would need to be addressed at a national level with policy change providing more benefits to asylum seekers. Interestingly, from the nominal group technique, the second most important barrier perceived by the stakeholders was cultural barriers. Although this was raised within the interviews, this wasn’t discussed by participants as much as the other barriers that were identified.

## Conclusions

Evidence suggests that moderate physical activity and/or exercise can improve mental health and wellbeing. For groups such as asylum seekers, who live in challenging circumstances, encouraging participation in such activities could be a priority for public health policy. This study has identified there are considerable barriers that influence uptake and adherence to physical activity and/or exercise for asylum seekers, yet, this is a group for whom the prevalence of mental health symptoms is high. Acting to overcome such barriers through public health interventions could encourage an increase in physical activity which may in turn reduce the breadth and depth of mental health needs of this group. We have found that asylum seekers are more than cognisant of the health benefits of physical activity and exercise, and many are motivated to increase their levels. Developing an intervention to ensure that they are aware of what is accessible locally could be an important stepping stone to increasing participation, improving health and wellbeing in this vulnerable group.
